# Characteristics and experiences of lactating women with measured low milk production

**DOI:** 10.1186/s13006-025-00753-1

**Published:** 2025-08-16

**Authors:** Theresia Margaretha Nicole Manshanden, Sarah Gallo Abelha, Joost Velzel, Jacki Louise McEachran, Donna Tracy Geddes, Sharon Lisa Perrella

**Affiliations:** 1 Department of Obstetrics & Gynaecology, Northwest Clinics, Alkmaar, Netherlands; 2 School of Molecular Sciences, The University of Western Australia, Crawley, WA 6009 Australia; 3 ABREAST Network, Perth, WA 6000 Australia; 4 UWA Centre for Human Lactation Research and Translation, Crawley, WA 6009 Australia

**Keywords:** Breastfeeding, Breast milk expression, Lactation, Human milk, Milk production

## Abstract

**Objective:**

This study aimed to compare the characteristics and experiences of women with measured low and normal 24 h milk production.

**Methods:**

We analysed data from a nested case-control study of 136 participants who measured their 24 h milk production within 1–6 months of birth and completed an online survey of lactation risk factors and experiences within 2 years of birth. The study was conducted between January 2020 and March 2024. 24 h milk production, calculated as the sum of all pre-post breastfeed and expression weights, was classified as low (< 600 mL) or normal milk production (≥ 600 mL). The prevalence of anatomical, endocrine/metabolic, pregnancy, birth complications and postpartum lactation risk factors was reported. Further, the experiences of participants that reported low milk production were described.

**Results:**

Low milk production was measured in 39 out of 136 participants (29%). Breast hypoplasia was more prevalent in this group (low milk production 13%; normal milk production 3%; *p* = 0.03). Of those with measured low milk production 21% perceived production was normal. In participants with measured normal production 28% had perceived low production. Formula use was more common among those with low milk production, and their infants had significantly lower weight-for-age z-scores despite similar birth weights. Qualitative data reflected the stress and effort expended in trying to increase milk production, and 10/26 (39%) rated lactation consultant support as most helpful in managing their milk production.

**Conclusions:**

Low milk production is a multifactorial and common concern, affecting nearly one in three breastfeeding women. While some contributing risk factors such as breast hypoplasia were identified, over half of the affected participants had not received an explanation from their healthcare provider. This underscores that low milk production is not always fully explainable or treatable, and highlights the need for personalized supportand further research to improve clinical assessment and effective management.

## Introduction

Many families do not meet the World Health Organization’s recommendation of exclusive breastfeeding for the first six months [1]. One of the most frequently cited reasons for early cessation of exclusive or any breastfeeding is self-reported insufficient milk production [2]. While the estimated prevalence of primary insufficient milk production is reported to be 10–15% [3, 4, 5], the prevalence of lactation ‘insufficiency’ as defined by individuals’ perceptions of ‘not having enough milk’ or ‘baby not satisfied with milk’ is much higher at 25% [6]. Successful breastfeeding depends on a complex interplay of physiological, behavioral and socioecological factors [2, 7]. Identification of the characteristics and experiences of those with insufficient milk production will support the development of tailored interventions to enhance breastfeeding support and outcomes.

There is no published definition of low milk production, yet it is important for both health care professionals and breastfeeding families, as adequate milk volume intake accounts forthe greatest variability of infant weight gain [8, 9]. While the average 24 h breast milk intake of infants aged 1–6 months is 750–800 mL/24 h, a range of 24 h production volumes is reported [8, 10]. The attainment of full milk production is contingent upon early and frequent milk removal in the postpartum period [11, 12]. However a number of factors may result in low milk production [7, 10, 13, 14], and may be categorised as extrinsic or intrinsic. Extrinsic factors, which limit milk removal from the breast, include infant anomalies or conditions that impair effective infant sucking, infant separation, infrequent breastfeeds or altered breast anatomy due to surgery [7, 10]. Intrinsic factors that limit milk production include endocrine conditions and aberrant or insufficient glandular tissue development such as breast hypoplasia, insulin resistance, obesity, thyroid disease, pregnancy complications and hypertension disorders [7, 10, 15]. Health professionals do not routinely assess individuals for lactation risk factors and there are gaps in knowledge regarding the management of subsequent low milk production [16]. Individuals who are unable to exclusively breastfeed may struggle with accepting low milk production and expend a lot of time and energy on interventions to increase their supply, with negative impacts on their mental health [17, 18].

Beyond the physiological and clinical aspects, breastfeeding also carries significant political, social, cultural, and existential meaning [2, 19, 20]. For many women, low milk production is not only a biological issue but closely tied to societal expectations, cultural ideals of motherhood, and personal identity. Feelings of guilt, inadequacy, or failure are common when breastfeeding goals are unmet, especially in environments where breastfeeding is seen as a moral obligation [21]. Moreover, breastfeeding is shaped by broader structural and cultural factors, including healthcare support, family roles, workplace policies, and norms around public and prolonged breastfeeding [22]. Recognizing this complexity is crucial to understanding both the impact and experience of low milk supply.

This study aimed to describe the characteristics of lactating women with measured low and normal 24 h milk productions including the prevalence of identified lactation risk factors. Further, the experiences of women with perceived low milk supply were explored.

## Materials and methods

This nested case-control study was conducted in Western Australia. Women aged ≥ 18 years who had birthed a singleton infant at term and had measured their milk production for one 24 h period between January 2020 to March 2024 when the infant was 1–6 months of age were invited by email to participate in an online survey. Potential participants were provided with written study information and the opportunity to receive further study information by contacting the researchers. After providing written informed consent participants used an electronic link to access the survey. While the overall data collection period spanned five years, all participants included in this analysis completed the survey within two years of their individual milk production measurement. Low milk production was defined as < 600 mL/ 24 h, based on a reported mean 24 h milk production at 1–6 months postpartum of 788 mL ± 169 [8].

*24 h milk production measurement*: Data were obtained from women that had participated in previous and continuing breastfeeding studies that include 24 h milk production measurement within 1–6 months of birth. These studies included a longitudinal cohort study where milk production was measured at 3, 9 and 12 months [23], and ongoing 24 h milk production studies that examine milk production and composition and maternal characteristics [24, 25]. When a participant had completed more than one milk production measurement, we selected the first measurement that was completed within 1–6 months of birth. Participants were provided with written and verbal instructions on how to complete the measurements in their own homes. A set of digital infant scales (BabyWeigh™; Medela Inc., McHenry, IL, resolution, 2 g accuracy, ± 0.034%) was provided. Prior to commencing the measurements, participants recorded their infant’s naked weight and completed a background questionnaire to provide details of demographics, health, pregnancy, birth, and lactation history. For one 24 h period infants were weighed before and after every breastfeed, and (if applicable) every bottle feed. For each bottle feed, participants recorded the type of nutrition (their own expressed breast milk, donor human milk or commercial milk formula (formula)). Participants who expressed were instructed to weigh the milk collection bottle before and after every breast expression. The pre-post weight differences for feeds and expressions were calculated and as 1 mL human milk weighs 1.03 g we considered this to be equivalent and reported results as mL [26]. The 24 h milk production was determined using the formula below, whereby *N* is the total number of breastfeeds and expressions, *v*_*i*_ is the volume of each breastfeed or expression, and *T* is the time that elapsed from the end of the first breastfeed until the end of the last breastfeed.

$$\:MP=\sum\:_{i=2}^{N}{v}_{i}\frac{24}{T}$$

That is, the calculation of 24 h milk production included all milk volumes removed from the breast through breastfeeding and breast expression. Infant feeds that are supplementary to direct breastfeeding during the 24 h period e.g. intake of mother’s expressed breast milk, donor human milk and formula, are not included in the calculation of 24 h milk production.

*Survey*: After providing informed consent, participants completed an online survey about their breastfeeding experience and gave permission for their background questionnairedata (provided at the time of 24 h milk production measurement) to be linked to their survey responses. The survey consisted of 33 items including both closed and open-ended questions relating to breastfeeding and lactation including current feeding status and date of breastfeeding cessation. We assessed self-reported anatomical risk factors for low milk production such as prior breast surgery. Breast hypoplasia was assessed by asking: “Has a health professional ever told you that you have breast hypoplasia or insufficient glandular tissue?”. The participants prior objectively measured 24-h milk production measurement was not referred to during this study. Participants were asked to indicate any breastfeeding concerns they had experienced during the current lactation. For those who reported concerns about low milk supply (that we will refer to as “perceived low milk supply”), regardless of prior measured milk production volume, the survey was expanded to include questions about their experience of breastfeeding, sources of support and strategies used to manage low milk supply. Items relating to low milk supply included closed questions, ordinal questions, and a qualitative component with open questions to report experiences. Participants without reported milk supply concerns did not complete this expanded section of the survey. Electronic consent and data collection were managed through REDCap [27].

*Statistical analysis*: Participant characteristics were examined using descriptive statistics with binary outcomes presented as counts and percentages, and continuous data presented as median and 25th and 75th quartiles. Differences between ‘low’ and ‘normal’ milk production group characteristics were compared with χ2 test, unpaired t-test, or non-parametric tests as appropriate. Infant weight-for-age z-scores (WAZ), that express an infant’s weight relative to the reference population’s expected weight for their age and sex as the number of standard deviations below or above the mean, were calculated for birth weight and weight at time of milk production measurement. A *p*-value of < 0.05 was considered statistically significant.

*Qualitative analysis*: Participants’ written responses to questions about their experiences of perceived low milk supply, including support and advice found to be helpful in managing low supply and experiences of completing the 24 h milk profile, were analysed using content analysis. To increase validity, two researchers (SLP and SGA) performed the analysis separately before discussing the results and obtaining consensus [28]. They independently familiarized themselves with the qualitative data by reading all responses before coding the data by grouping words, sentences and paragraphs with similar meanings to identify concepts. Transcripts were then re-read to ensure that all content relevant to the study aims were included. The researchers then examined the codes for themes and categories and worked together to achieve consensus on findings of participants’ perceptions of the challenges of low milk supply and helpful aspects of its management. Participant quotes used to illustrate the findings were identified by the participant’s study identification code and measured 24 h milk production (e.g. #25000, xxxml) with those < 600mL/24 h in the measured low milk production group.

Both the 24 h milk production study (RA/4/20/6134) and the study of characteristics of women with measured low and normal milk production (NMP) (2022/ET000356) were approved by the Human Research Ethics Committee of The University of Western Australia. All participants provided written informed consent.

## Results

**Fig. 1:**
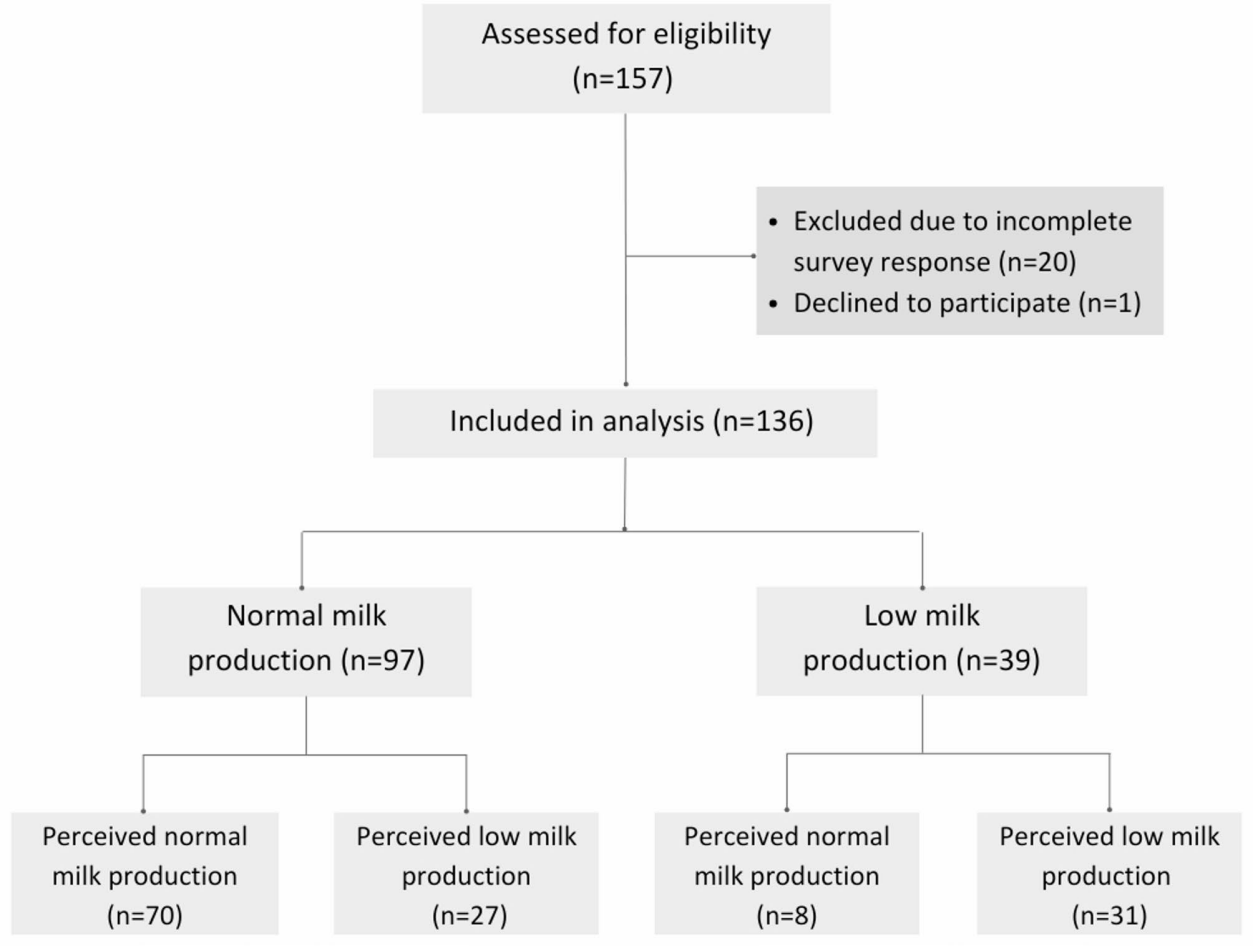
Study flow diagram

Of the 157 participants who completed measurement of their milk production and the subsequent study survey, *n* = 136 met the study inclusion criteria (Fig. 1). Participant characteristics, shown in Table 1, were recorded as part of a prior breastfeeding study during which milk production was measured. Participants completed the 24 h milk production measurement at 13 (9, 16) weeks after birth (Table 2), and completed the subsequent study survey at 53 (36, 90) weeks after birth (Table 3). The shortest time interval from 24 h milk production measurement to survey completion was 2.4 weeks. Infant feeding characteristics and weight at the time of 24-hour milk production measurement, and infant birth weight, are presented in Table 2 for participants with measured low and normal milk production. Overall the median (IQR) 24 h milk production was 731 mL (IQR 584–842), with a low milk production measured in 39/136 (29%). While breastfeeding frequencies were similar between groups, the median 24 h volume of formula feeds was significantly higher for the measured low production group (low 158 mL (IQR 0–363 mL); normal 0 mL (IQR 0–0); *p* < 0.001). Few participants reported performing breast expression at the time of 24 h milk production measurement (normal milk production group: *n* = 20 (23%); measured low milk production group: *n* = 9 (20%), *p* = 0.62).

**Table 1:** Participant characteristics of study sample and sub-groups with measured normal and low milk production as reported at survey completion. Data reported as median (Q1, Q3), mean ± standard deviation or n (%)

	ALL (*N* = 136)	NMP (*n* = 97)	LMP (*n* = 39)	*P*-value
Pre-pregnancy BMI BMI ≥ 30.0 (obese)	24.5 (22.0, 29.4) 14 (10%)	23.8 (22.0, 29.4) 10 (10%)	25.1 (22.8, 28.8) 4 (10%)	0.54 0.99
Primiparous	87 (64%)	61 (63%)	26 (67%)	0.68
Birth gestation (weeks)	39.1 ± 3.0	39.0 ± 3.5	39.2 ± 1.2	0.53
Vaginal birth	84 (62%)	61 (63%)	23 (59%)	0.67
Intended BF duration (months)	12 (12, 18)	12 (12,18)	12 (6, 12)	0.045
≤6 months	30 (22%)	20 (21%)	10 (26%)	0.52
>6 - ≤12 months	62 (46%)	39 (40%)	23 (59%)	0.047
>12 months	44 (32%)	38 (39%)	6 (15%)	0.007
Previous BF duration (months)^*^	14 (8, 23)	14 (9, 22)	12 (3, 29)	0.36
**Pre-existing anatomical lactation risk factors**				
Nipple surgery	1 (1%)	1 (1%)	0	1.00
Breast augmentation surgery	3 (2%)	3 (3%)	0	0.56
Nipple piercing	10 (7%)	7 (7%)	3 (8%)	0.92
Hypoplasia	8 (6%)	3 (3%)	5 (13%)	0.03
No breast growth in pregnancy	36 (26%)	23 (24%)	13 (33%)	0.25
**Pre-existing endocrine / metabolic lactation risk factors**				
Polycystic ovary syndrome	15 (11%)	11 (11%)	4 (10%)	0.85
Diabetes mellitus Type 1 or 2	2 (1%)	2 (2%)	0	1.00
Insulin resistance	3 (2%)	3 (3%)	0	0.56
Hyperthyroidism	1 (1%)	1 (1%)	0	1.00
Hypothyroidism	9 (7%)	8 (8%)	1 (3%)	0.23
**Pregnancy complications**				
Assisted reproduction	17 (13%)	10 (10%)	7 (18%)	0.22
Gestational diabetes mellitus	30 (22%)	19 (19%)	11 (28%)	0.27
Gestational hypertension	13 (10%)	11 (11%)	2 (5%)	0.27
Pre-eclampsia	7 (5%)	5 (5%)	2 (5%)	1.00
Fetal growth restriction	6 (4%)	3 (3%)	3 (8%)	0.24
**Postpartum lactation risk factors**				
Postpartum hemorrhage	20 (15%)	13 (13%)	7 (18%)	0.50
Postpartum hypertension	9 (7%)	7 (7%)	2 (5%)	0.66
Neonatal nursery admission	20 (15%)	14 (14%)	6 (15%)	0.89

*Only multiparous included *n* = 48. NMP = normal milk production; LMP = low milk production; BMI = body mass index; BF = breastfeeding

**Table 2:** 24 h milk production, milk intake, breastfeeding characteristics and birth and current infant weights as reported at time of milk production measurement. Data reported as median (Q1, Q3) or n (%)

24 h MP characteristics	ALL (*N* = 136)	NMP (*N* = 97)	LMP (*N* = 39)	*P*-Value
MP (mL)	731 (584, 842)	786 (711, 897)	444 (320, 543)	< 0.001
Formula intake (mL)	0 (0, 0)	0 (0, 0)^*^	158 (IQR 0-363)	< 0.001
BF frequency	12 (9, 15)	13 (10, 15)	11 (9, 14)	0.56
Expression frequency	0 (0, 0)	0 (0, 0)^* *^	0 (0, 5)	0.95
Total milk removal freq	14 (11, 16)	13 (11, 16)	14 (10, 17)	0.82
Infant weight				
Birth weight (g)	3335 (3115, 4822)	3355 (3160, 3640)	3270 (2983, 3628)	0.29
WAZ at birth	0.5 (0.0, 1.2)	0.6 (0.1, 1.2)	0.4 (0.0, 0.9)	0.59
Age at 24 h MP (weeks)	13 (9, 16)	13 (8, 15)	13 (10, 18)	0.34
Weight at 24 h MP (g)	*n* = 123 5716 (3636, 6363)	*n* = 87 5942 (4968, 6586)	*n* = 36 5310 (4586, 6057)	0.02
WAZ at 24 h MP	*n* = 123 -0.4 (-0.9, 0.2)	*n* = 87 -0.2 (-0.7, 0.3)	*n* = 36 -0.7 (-1.2,0.3)	< 0.001

*n = 10 reported formula feeding volumes. **n = 20 reported expression frequency. NMP = normal milk production; LMP = low milk production; MP = milk production measurement; BF = breastfeeding; WAZ = weight-for-age Z-score

**Table 3:** Breastfeeding characteristics and infant weight at the time of survey completion. Data reported as median (Q1, Q3) or n (%)

BF characteristics	ALL (*N* = 136)	NMP (*N* = 97)	LMP (*N* = 39)	*P*-Value
Age at survey (weeks)	53 (36, 90)	53 (35, 90)	54 (39, 90)	0.07
Still BF	51 (29, 81)	52 (34, 82)	44 (24, 61	0.31
Ceased BF	59 (39, 116)	114 (37, 138)	51 (40, 59)	0.01
BF ceased	48 (35%)	27 (28%)	21 (54%)	0.004
BF duration (months)	10.5 (6, 16)	13 (6.5, 18)	6 (5, 10)	0.029
Ceased earlier than hoped	28 (58%)	13 (48%)	15 (71%)	0.001
Reasons for ceasing BF	*n* = 36	*n* = 15	*n* = 21	
Nipple pain	3 (8%)	1 (7%)	2 (10%)	0.14
Mastitis	4 (11%)	3 (20%)	1 (5%)	0.87
Low milk supply	16 (44%)	5 (33%)	11 (52%)	< 0.001
Return to work	3 (8%)	0 (0%)	3 (14%)	0.02
Other*	10 (28%)	6 (40%)	4 (19%)	0.41

*n = 6 cited pregnancy / trying to achieve pregnancy as a reason for stopping BF. NMP = normal milk production; LMP = low milk production; BF = breastfeeding

Infant age at measurement of 24 h milk production was 13 (IQR 9–16) weeks, and was similar between groups (*p* = 0.34). Infants in the measured low milk production group had significantly lower weight and WAZ at measurement of 24 h milk production (Table 2), despite no significant differences in infant weights and WAZ at birth between groups (Table 2). Supplementary formula use was reported by 22/39 (56%) of the measured low milk production group.

The median intended breastfeeding duration was 12 months in both groups; however, the interquartile range was significantly shorter in the measured low milk production group (6–12 months) compared to the normal milk production group (12–18 months; *p* = 0.045). The prevalence of identified lactation risk factors is reported in Table 1, with self-reported breast hypoplasia more prevalent in those with measured low milk production (13%) than in those with normal milk production (3%; *p* = 0.03). The prevalence of other anatomical breast factors, endocrine/metabolic factors, pregnancy and birth complications and postpartum risk factors were comparable between the two groups (Table 1).

Of the participants no longer breastfeeding at the time of survey completion, a higher proportion of those with measured low milk production had stopped earlier than planned, with most stopping before 6 months postpartum. Perceived low milk supply was a more commonly reported concern and reason for weaning in those with measured low milk production than for those with normal milk production (Table 3). The prevalence of other reported breastfeeding concerns including nipple pain were similar between groups.

Despite having measured normal milk production, *n* = 27 (28%) participants reported that they had experienced concerns about insufficient milk supply during that lactation. Therefore we further investigated sub-groups of participants that ‘measured low milk production and perceived low milk supply’ (*n* = 31, 80%) and ‘measured normal and perceived low milk supply’ (Fig. 1). While characteristics of these perceived low milk supply sub-groups were not different, a small number in each sub-group self-reported breast hypoplasia, which was not reported for groups that perceived normal milk supply (Table 4).

**Table 4:** Characteristics of groups with measured and perceived low and normal milk production as reported at survey completion. Data reported as median (Q1, Q3), mean ± standard deviation or n (%)

	Measured LMP < 600 mL/24 h (*N* = 39)				*P*-value	Measured NMP ≥ 600 mL/24 h (*N* = 97)			*P*-value
	Perceived NMP (*n* = 8)	Perceived LMP (*n* = 31)				Perceived NMP (*n* = 70)	Perceived LMP (*n* = 27)		
Pre-pregnancy BMI	25.0 (23.3–27.5)	25.1 (22.8–28.8)			1.00	24.0 (22.5–29.8)	22.9 (21.1–26.9)		0.09
Pre-pregnancy weight (kg)	68 (64, 76)	68 (62, 83)			1.00	69 (62, 85)	69 (57, 75)		0.21
Primiparous	4 (50%)	22 (71%)			0.26	40 (57%)	21 (78%)		0.06
Birth gestation (weeks)	39.6 ± 1.4	39.1 ± 1.1			0.83	39.3 ± 1.2	38.3 ± 6.4		0.38
24 h MP (mL)	569 (412, 577)	435 (292, 504)			0.10	791 (714, 897)	767 (715, 882)		0.74
Vaginal birth	6 (75%)	17 (56%)			0.30	43 (62%)	18 (67%)		0.63
Intended BF duration	12 (12, 12)	12 (6, 12)			0.55	12 (12, 24)	12 (6, 12)		0.02
≤6 months	1 (13%)	9 (29%)			0.34	11 (16%)	9 (33%)		0.06
>6 - ≤12 months	6 (75%)	17 (55%)			0.30	27 (39%)	12 (44%)		0.60
>12 months	1 (13%)	5 (16%)			0.80	32 (46%)	6 (22%)		0.034
Previous BF duration (months)*	20.5 (10, 29)	10 (2, 13.5)			0.32	18 (12, 23.5)	11.5 (5.5, 13.8)		0.60
**Anatomical lactation risk factors**									
Nipple piercing	0			3 (10%)	1.00	4 (6%)	3 (11%)		0.36
Hypoplasia	0			5 (16%)	0.56	0	3 (11%)		0.005
No breast growth in pregnancy	1 (13%)			12 (39%)	0.16	15 (21%)	8 (30%)		0.40
**Endocrine / metabolic lactation risk factors**									
PCOS	1 (13%)			3 (10%)	0.82	7 (10%)	4 (15%)		0.50
Insulin resistance	0			0	NA	3 (4%)	0		0.56
Hypothyroidism	0			1 (3%)	1.00	5 (7%)	3 (11%)		0.53
**Pregnancy complications**									
Assisted reproduction	0			7 (23%)	0.31	6 (9%)	4 (15%)		0.37
GDM	1 (13%)			10 (32%)	0.27	14 (20%)	5 (19%)		0.87
Gestational hypertension	0			2 (6%)	1.00	8 (11%)	3 (11%)		0.97
Pre-eclampsia	0			2 (6%)	1.00	4 (6%)	1 (4%)		0.69
Fetal growth restriction	0			3 (10%)	1.00	2 (3%)	1 (4%)		0.83
**Postpartum lactation risk factors**									
PPH	2 (25%)		5 (16%)		0.56	9 (13%)		4 (15%)	0.80
Hypertension	0		2 (6%)		1.00	5 (7%)		2 (8%)	0.96
NNU admission	1 (13%)		5 (16%)		0.80	12 (17%)		2 (8%)	0.22

*Only multiparous included *n* = 48. LMP = low milk production; NMP = normal milk production; BMI = body mass index; BF = breastfeeding; PCOS = polycystic ovary syndrome; GDM = gestational diabetes mellitus; PPH = postpartum hemorrhage; NNU = neonatal unit

Most participants that reported low milk supply were first aware of this in the first month after birth. Approximately half first identifed low milk supply within 2 weeks postpartum (48% measured and perceived low milk supply; 50% measured normal and perceived low milk supply), with lower proportions first identifing it at 2–4 weeks postpartum (15% measured and perceived low milk supply; 19% measured normal and perceived low milk supply). There was no statistically significant difference in the timing of first awareness of low milk supply between groups (*p* = 0.77).

Of those with measured low milk supply, 22% reported being informed by a health care provider about potential lactation risk factors for low milk production before birth, and 56% indicated they had not received an explanation for their low milk supply by their health care provider. However, this does not necessarily imply the absence of contributing factors, as some individuals may not have sought care or received a comprehensive evaluation. The most frequently cited sources of helpful breastfeeding support were the participant’s partner (88%), lactation consultant accessed after hospital discharge (100%), and the Australian Breastfeeding Association (73%). Lower proportions rated the support of a medical doctor as helpful (obstetrician 53%, family doctor 45% and paediatrician 23%).

**Fig. 2:**
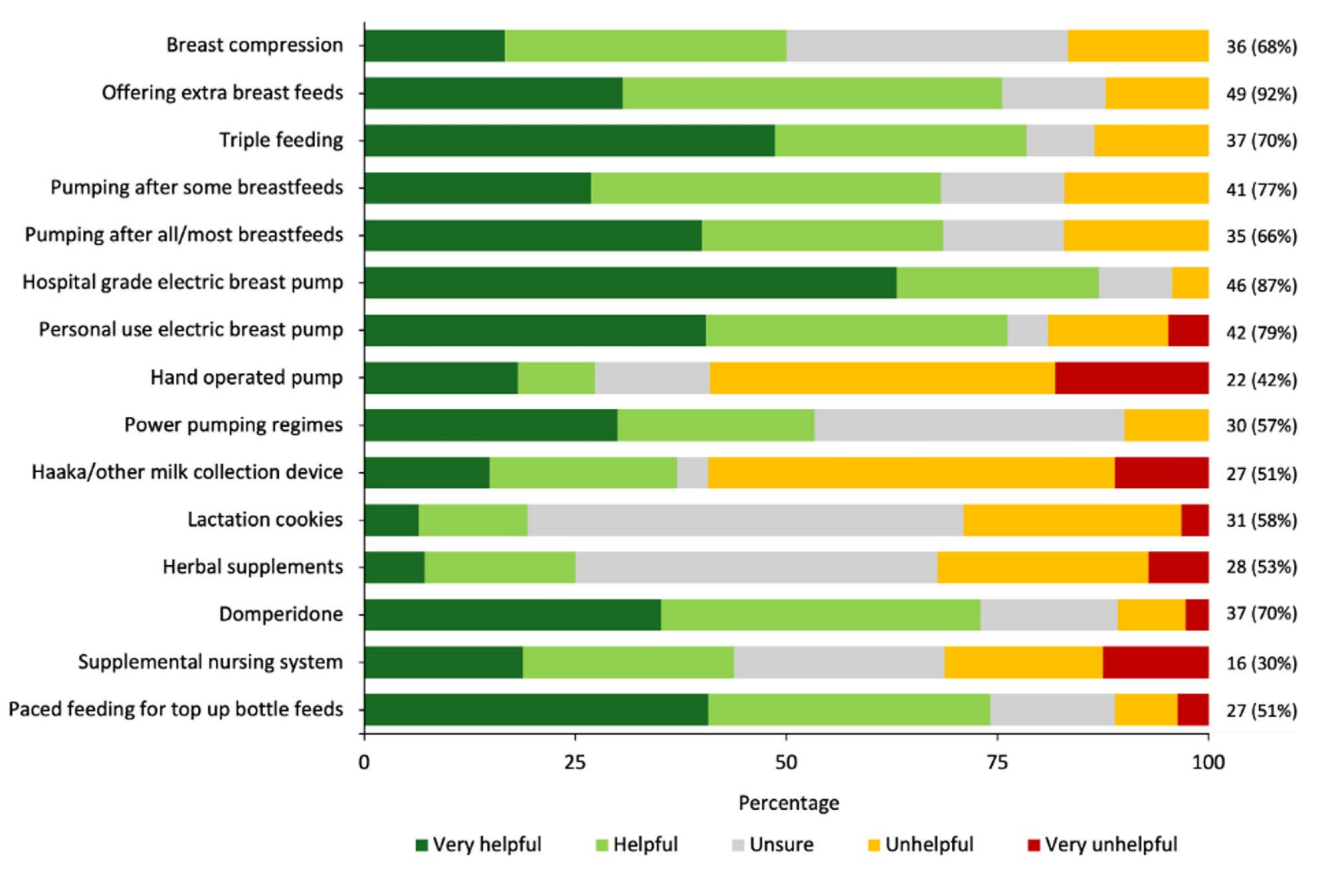
Strategies used to manage low milk supply and ratings of their helpfulness in participants that perceived insufficient milk supply during their lactation (*n* = 53)

The various strategies used to manage low milk supply and their ratings of helpfulness are shown in Fig. 2. The most frequently utilized strategies were; offering extra breastfeeds (92%), and breast expression using a hospital-grade electric breast pump (87%) or personal electric breast pump (79%). Overall, use of a hospital-grade electric breast pump was rated as the most helpful (*n* = 40, 95%), followed by triple feeding (*n* = 29, 78%) which entails breastfeeding, expressing milk and giving supplementary feeds of expressed milk and/or formula at every feed. Participants with measured normal milk production and perceived low milk supply were significantly more likely to rate certain strategies as helpful or very helpful compared to those with measured low milk production and perceived low milk supply. Specifically, a higher proportion rated pumping after all or most breastfeeds as helpful (normal 93% vs. low 52%, *p* = 0.012), triple feeding (normal 94% vs. low 63%, *p* = 0.021), and use of a personal use electric breast pump (normal 91% vs. 60%, *p* = 0.019). Non-pharmacological galactagogues such as lactation cookies and herbal supplements were infrequently reported to be helpful or very helpful. For the other reported strategies, no statistically significant differences in perceived helpfulness were observed between the two groups.

Of those who reported perceived low milk supply, 27/41 participants reflected on what they wish they had known or done differently in relation to having low milk supply. The qualitative data mostly highlighted the stress and efforts invested in trying to increase milk production. One participant recalled “*Over 6 months*,* I did 925 pumping sessions. That’s a lot of hours to be sitting down*,* not moving and not being able to look after myself or the baby”* [#23081, 555 ml] and another wished she had *“a better plan instead of the stress that started with so much cluster feeding and the stress about it all in the early days and the ridiculous triple feeding regime”* [#23079, 92 ml]. Nine participants wished they had known more about breastfeeding, or that low milk production was even a possibility. While seven participants regretted not seeking professional help earlier, twelve lamented following professional advice regarding formula supplementation, limiting of breastfeeding frequency and/or skin to skin contact.

Of 26 participants who provided feedback on support and advice that was most helpful in managing low milk supply, 10 mentioned the support of a lactation consultant. One participant wrote: *“I found the lactation consultant most helpful. Even though I felt defeated when I had low supply and had to give formula*,* she assured me I was doing the right thing and that my baby was growing and getting fed’ [#21031*,* 158 ml].* Another said: *“…everything about low supply is hard but having a good LC made me feel so much better.” [22057*,* 406 ml]* Partner and family support were frequently cited, with one participant reporting “*My family could look after the baby while I pumped. Without them I would have had to stop much earlier.”* [*23081*,* 274 ml].*

Some reported the most effective strategy to be frequent milk removal through breastfeeding and/or expressing, and some specifically stated that an understanding of the physiology of milk production was very useful in guiding their strategies. A few participants cited peer groups, Instagram and Facebook groups as being helpful, although one explained that it could be a double-edged sword in that it was helpful as long as breastfeeding was going well but *“…triggering when things didn’t work.”* [20200, 531 ml]. Some participants reported that “nothing helped” and emotional distress was evident with one woman stating *“it was the hardest time of my life*,* I felt like a complete failure and it felt like every thing was a reminded (sic.) of low milk and my inability to feed my child*.” [20137, 219 ml].

Dichotomous experiences of measuring 24 h milk production were described. While 10/22 cited challenges of sleep disruption or managing the test weighing while triple feeding, another 10/22 reported benefits indicating that the results were validating and reassuring with regard to guiding formula supplementation volumes.

## Discussion

In this nested case-control study the prevalence of perceived low milk supply was 43%. Perceived low milk supply was reported by 28% people with a measured normal milk production and 79% with measured low milk production, with slower infant growth observed in the measured low milk production group. Breast hypoplasia was reported by a small number of participants with perceived low milk supply and none with measured normal milk production. For many the experience of perceived low milk supply was stressful, characterized by unhelpful professional advice and and time consuming management strategies.

Breastfeeding is the unequivocal gold standard in infant nutrition, offering unparalleled benefits for both mother and child. However, the reality remains that not everyone can exclusively breastfeed due to multifaceted factors [4, 10, 29]. An understanding of the complex interplay of extrinsic and intrinsic lactation factors is imperative in addressing disparities in breastfeeding rates [7, 10]. In our sample, lactation risk factors were present in participants with both normal and low measured milk production, and the prevalence of reported breast hypoplasia was 6% (Table 1).

Breast hypoplasia is associated with atypical breast features such as breast asymmetry, a wide intermammary width, an absence of breast growth during pregnancy, and subsequent low milk production [30, 31]. While 6% participants in our study sample reported breast hypoplasia, its prevalence has not been reported in larger populations, and earlier reports have suggested it is a rare condition [32]. Absence of breast growth in pregnancy was reported by 26% of our total sample.Among participants with measured low milk production, 33% reported no breast growth, compared to 24% in the measured normal milk production group (Tables 1 and 4). This finding approximates with the findings of Neifert et al. who reported 24% of primiparous women had minimal breast growth, of which 25% had low infant weight gain in the first 3 weeks after birth [5]. Similarly, the prevalence of absent breast growth in pregnancy is higher in lactating women with perceived low milk supply [31]. Interestingly, the prevalence of breast hypoplasia was higher among participants with perceived low milk supply in both measured low and normal milk production groups. This may reflect a bi-directional relationship: concerns about milk supply may bias the perception of hypoplasia, or conversely, perceived hypoplasia may lead to greater concerns about milk supply. Due to the retrospective design and reliance on self-report, we are unable to determine the direction or causality of this association. Our study results indicate that breast hypoplasia that has been identified by a health care provider is not a ‘rare’ anomaly, and larger cohort studies are needed to accurately determine the nature and prevalence of breast hypoplasia [33].

In this study 43% of participants reported that they had perceived low milk supply at some point during their breastfeeding duration, of which 13.8% identified as having breast hypoplasia (Table 4). A recent systematic review reported a wide range of rates of perceived insufficient milk supply across different time points and settings with a prevalence of 25% at less than one week postpartum that reduced to 15% at four to six months postpartum [6]. Our reported prevalence of both perceived low milk supply and measured low milk production may not be representative of the wider population, as the study participants were self-selected to breastfeeding studies involving 24 h milk production measurement, were motivated to breastfeed as shown by the long intended breastfeeding duration, and pregnancy complications associated with suboptimal lactation outcomes were over-represented in our sample.

With the cutoff point of 600 mL/24 h, the prevalence of 29% objectively measured low milk production was lower than the 43% perceived low milk supply (Tables 1 and 4). Several factors may explain this discrepancy. For example, participants were asked to indicate if they had encountered low milk supply concerns during their breastfeeding duration, which was not necessarily at the specific timepoint at which milk production was measured. Also, perceptions of milk supply are influenced by breastfeeding confidence and knowledge, with infant crying often interpreted as a sign of insufficient milk that may not be correct [6]. Lastly, as a wide range of normal 24 h milk intake volumes are observed in the fully breastfed infant [26], criteria additional to a volume threshold such as infant growth would account for individual infant volume requirements. Our results have shown that despite comparable infant birth weights and WAZ at birth between the groups, at the time of 24 h milk production measurement low milk production group infants were of similar age but had significantly lower weights and WAZ, on average 632 g lower, than those of the normal milk production group (Table 2). This highlights a need for further examination of low milk production and infant growth with consideration of supplementation, infant health and other factors that impact infant growth. Refinement of the definition of low milk production can be progressed with a longitudinal prospective study design, objective measurements of milk production and intake, infant sex, health and anthropometric data.

Participants engaged multiple strategies to manage their perceived low milk supply, and indicated this was challenging and not always effective. Different types and frequencies of breast pump use were reported as helpful or very helpful, with use of a hospital grade breast pump receiving the highest rating (Fig. 2). Indeed frequent and adequate extraction of milk from the breast is a common strategy to increase milk production [11, 34], with simultaneous breast expression using an electric breast pump shown to be more effective than hand expression [35, 36]. Triple feeding was perceived to be helpful by 78% of participants, and has been reported to be useful in overcoming barriers for ineffective breastfeeding [37]. However this strategy is time consuming and associated with negative breastfeeding experiences, as people can become fixated on their milk production [18, 38]. Studies of effectiveness and experiences of pumping to increase milk production after birth at term are lacking. While increasing the frequency and adequacy of milk removal through breastfeeding and/or breast expression addresses the autocrine control of milk production [11, 34], this strategy likely cannot resolve anatomical, endocrine or genetic abberrations that impair milk production.

The use of galactogogues such as ‘lactation cookies’, herbal supplements, and medications was reported (Fig. 2). Ratings of helpfulness of nonpharmacological galactagogues were low, which is congruent with inconsistent published findings on their effectiveness in treating low milk supply [39]. The majority of participants that had used domperidone indicated it was helpful. Domperidone is the most extensively studied pharmacological galactagogue, particularly within the preterm population where women are at increased risk of low milk production [40]. However, the generalizability of domperidone’s use remains unclear, and serious adverse effects can also occur [41]. Given the variable effectiveness of galactatogues and practical strategies to increase milk production, there is an urgent need for accessible clinical measures of lactation, that may include 24 h milk production measurements and biochemical tests to guide the management and care of families with perceived low milk supply.

Besides strategies to increase milk production, our study highlights the significant impacts of professional and social support on managing low milk supply. Of those who reported on valuable sources of support several cited their lactation consultant, and most mentioned the crucial role of partner and family support. Partners are not routinely included in breastfeeding education, yet recent research indicates that partners want to learn from health care providers about common breastfeeding challenges, their management, and how best to support their partner [42]. A future challenge will be to incorporate partners and family into breastfeeding education programs, as this inclusion may further enhance the wellbeing and support of families managing low milk supply.

Participants’ qualitative feedback highlighted the emotional burden and workload associated with managing low milk supply. The reported disappointment, sadness, worries about adequate infant weight gain and negative emotions associated with using commercial milk formula supplementation were similar to those described in a recent Irish study with nine first-time parents and perceived low milk supply. Balancing infant care with time intensive breastfeeding, breast expression and supplementary feeding regimes was a common struggle, and some alluded to their experience as traumatic [18, 38]. While more extended qualitative responses were provided by participants with measured low milk production, further research is needed to investigate the degree of stress associated with perceived low milk supply regardless of the measured milk production volume. The practical and emotional support of the family, and lactation consultant support were integral for many, while a lack of breastfeeding knowledge and professional help made the experience more difficult. These insights indicate a need for ongoing education and support for families experiencing LMP.

The main limitation of this study is its retrospective design, which prevents us from determining whether potential contributing mechanisms influenced milk production measurements and perceptions. Combined with the relatively small sample size, these limitations mean that the study is best interpreted as descriptive in nature, with limited ability to detect statistically significant differences or draw conclusions regarding the prevalence of lactation risk factors in women with measured low and normal milk production. Additionally, the use of a single 24 h milk production measurement provides only a momentary view of lactation that does not reflect the entire lactation experience. For example, some participants with normal measured milk production may have previously experienced low milk supply. We emphasize that measured low milk production reflects an outcome at a specific time point and does not in itself indicate whether low milk production results from intrinsic physiological insufficiency or modifiable external factors such as feeding practices, infant behavior, or lactation management. This descriptive study is intended to inform the design of a future prospective study that is adequately powered to characterize differences between groups with measured low and normal milk production.

## Conclusion

This study highlights that perceived low milk supply and measured low milk production may be more prevalent than commonly thought. In addition, this study indicates a higher prevalence of breast hypoplasia in women with measured low 24 h milk production, and there are time and emotional burdens associated with managing low milk production. Further research is needed to better understand the multiple factors that can impact milk production, and the education and support needs of those with low milk supply.

## Data Availability

The data that support the findings of this study are not openly available due to reasons of sensitivity and are available from the corresponding author upon reasonable request. Data are located in the controlled access Institutional Research Data Store at The University of Western Australia.
